# Increased Frequency of* Pfdhps* A581G Mutation in* Plasmodium falciparum* Isolates from Gabonese HIV-Infected Individuals

**DOI:** 10.1155/2019/9523259

**Published:** 2019-05-09

**Authors:** Jeanne Vanessa Koumba Lengongo, Yaye Dié Ndiaye, Marie Louise Tshibola Mbuyi, Jacques Mari Ndong Ngomo, Daouda Ndiaye, Marielle Karine Bouyou Akotet, Denise Patricia Mawili-Mboumba

**Affiliations:** ^1^Department of Parasitology Mycology, Faculty of Medicine, Université des Sciences de la Santé, Libreville BP4009, Gabon; ^2^Laboratory of Parasitology Mycology, Aristide le Dantec Hospital, Faculty of Medicine and Pharmacy, Cheikh Anta Diop University, 5005 Dakar, Senegal; ^3^The International Centers of Excellence for Malaria Research (ICEMR) Program, Dakar, Senegal

## Abstract

**Background:**

Studying malaria parasites cross resistance to sulfadoxine-pyrimethamine (SP) and trimethoprim-sulfamethoxazole (cotrimoxazole, CTX) is necessary in areas coendemic for malaria and HIV. Polymorphism and frequency of drug resistance molecular markers,* Pfdhfr *and* Pfdhps* genes have been assessed in* Plasmodium falciparum *isolates from HIV-infected adults, in Gabon.

**Materiel and Methods:**

A cross-sectional study was conducted in three HIV care and treatment centers, at Libreville, the capital city of Gabon and at Oyem and Koulamoutou, two rural cities between March 2015 and June 2016.* P. falciparum*-infected HIV adults were selected. Analysis of* Pfdhfr *and* Pfdhps *genes was performed using high resolution melting (HRM) technique.

**Results:**

* Pfdhps* A581G mutation was found in 23.5% (8/34) of the isolates. Triple* Pfdhfr *mutation (51I-59R-108N) was predominant (29.4%; n=10) while 17.6% (n=6) of the isolates carried a quadruple mutation* (Pfdhfr *51I-59R-108N +* Pfdhps *437G;* Pfdhfr *51I-108N +* Pfdhps *437G-*Pfdhps*581G;* Pfdhfr *51I-59R-108N +* Pfdhps *581G). Highly resistant genotype was detected in around 10% (n=3) of the isolates. The quintuple mutation (triple* Pfdhfr *51I-59R-108N and double* Pfdhps*437-581) was only found in isolates from two patients who did not use CTX. The most frequent haplotypes were those with a single mutation (**N**CNIAKA) (36%) and a quadruple mutation (**N**C**I**I**G**K**G**,** NRI**I**G**KA, and** NRI**IAK**G**). Mixed unknown genotypes were found at codon 164 in three isolates. Mixed genotypes were more frequent at codons 51 (23.5%; n=8) and 59 (20.5%; n=7) (*p*<0.01).

**Conclusion:**

* Pfdhps *A581G mutation as well as new combination of quintuple mutations is found for the first time in isolates from HIV-infected patients in Gabon in comparison to a previous study. The detection of these genotypes at a nonnegligible frequency underlines the need of a regular surveillance of antifolates drug resistance.

## 1. Introduction

*Plasmodium falciparum *infection constitutes a major public health problem in Africa where human immunodeficiency virus (HIV) infection is also frequent. According to the World Health Organization (WHO), by the end of 2016, 36.7 million people worldwide were HIV-infected and 25.5 million lived in sub-Saharan Africa [1]. Comorbidity due to both pathogens is not rare [2, 3]. Integrated care of patients with HIV infection and malaria in areas coendemic is now an international health challenge in order to control and reduce the burden of these infections.

In guiding control strategies, the monitoring of drug resistant malaria parasites spread is essential. Among the antimalarial drugs against those parasites develop resistance, antifolates, or sulfamides association is part of the panel. These molecules are highly used in primary healthcare structures; thus, their efficacy has to be regularly under surveillance. Indeed, sulfamides association such as sulfadoxine-pyrimethamine (SP) represents the cornerstone of malaria prevention for women and children. SP is used as an intermittent preventive treatment for prevention of malaria in pregnancy (IPTp-SP) and SP + Amodiaquine is the combination used for seasonal malaria chemoprophylaxis (SMC) in Africa [4]. Another sulfamide association, the cotrimoxazole (CTX, trimethoprim-sulfamethoxazole) is recommended for malaria prevention in endemic settings and opportunistic infections diseases such as pneumocystosis, toxoplasmosis, and isosporiasis among people living with HIV (PLHIV) [5]. Indeed, when used as a chemoprophylactic agent, CTX decreases by 90% the risk of malaria episodes and reduces the incidence of clinical malaria in PLHIV adults [2, 6–8]. IPTp-SP and CTX chemoprophylaxis coverage are high reaching 80% in Gabon [3, 9]. This leads to a substantial drug pressure on these DHFR (dihydrofolate reductase)/DHPS (dihydropteroate synthase) enzyme inhibitors with a high risk of selection and circulation of resistant parasites as observed elsewhere [10]. Indeed, resistance to SP is conferred by the cumulative single nucleotide polymorphism (SNP) mutation in* Pfdhfr* and* Pfdhps *genes encoding for enzymes of the malaria parasite folates pathway [11]. Mutations on* Pfdhfr *gene (S108N, C59R, N51I, and I164L) are related to an increasing resistance to pyrimethamine while those occurring on* Pfdhps* gene (A437G, K540E, A581G, and A613S/T) are associated with sulfadoxine resistance [12]. However, as reported for the I164L SNP, the A581G mutation, which leads to a highly resistant phenotype, is detected at low frequency in few countries of Africa [13, 14] while data from West or central Africa are scarce [15, 16]. The combination of the triple mutation of* Pfdhfr* gene (S108N, C59R, and N51I) is related to resistance to SP and when detected with the double* Pfdhps* (A437G + K540E) mutations, they collectively lead to a quintuple mutant “super-resistant” parasite described in Africa [17]. Moreover, this super-resistant genotype may alternatively carry* Pfdhps* 581G or* Pfdhfr* 164L mutation leading to “super resistant” parasite which predicts treatment failure in East Africa [17, 18]. It has been shown that* Pfdhfr* triple mutant generates high-level resistance to both trimethoprim and pyrimethamine [19]. Cross resistance to sulfadoxine and sulfamethoxazole, both sulfamides compounds acting on PFDHPS enzyme, should also be considered [20, 21]. All these data on antimalarial drug efficacy are recorded throughout the world in malaria endemic areas such as in Asia and/or Africa and are required for the monitoring of drug resistance spread. Such information may be available and shared via local or regional networks such as Worldwide Antimalarial Resistance Network (WWARN). However, few data on drug resistance molecular markers from malaria infected PLHIV population are available. The detection of these mutant genotypes and the increase of their frequency raises the fear of therapeutic failures and may hamper healthcare provided to the patients.

In Gabon, a nonnegligible frequency of* P. falciparum *submicroscopic infections has been found in PLHIV [22]. Such infections, if due to resistant parasite, may contribute to the spread of antimalarial drug resistance. Indeed, high frequencies of mutations on* Pfdhfr *and* Pfdhps *have been previously reported in* P. falciparum* isolates from SP users and no users [9, 23, 24]. Thus monitoring strategies of mutant parasites is required and should involve population such as HIV-infected individuals who can contribute to the parasite resistant reservoir as they frequently low carry density parasitemia [22]. In Gabon, no study describing drug resistance molecular markers was performed in isolates from PLHIV. Polymerase chain reaction (PCR) restriction fragment length polymorphism (RFLP) was always used for their detection in other populations. In this country, HIV prevalence ranges from 2 to 13% according to different regions [25]. Asymptomatic* P. falciparum *carriage was found in near 7% of PLHIV [3]. This study investigated the polymorphism and the frequency of drug resistance molecular markers,* Pfdhfr* and* Pfdhps* genes, using HRM in* P. falciparum *isolates from HIV-infected adults living in Gabon.

## 2. Patients and Methods

### 2.1. Study Areas and Population

**Figure 1:**
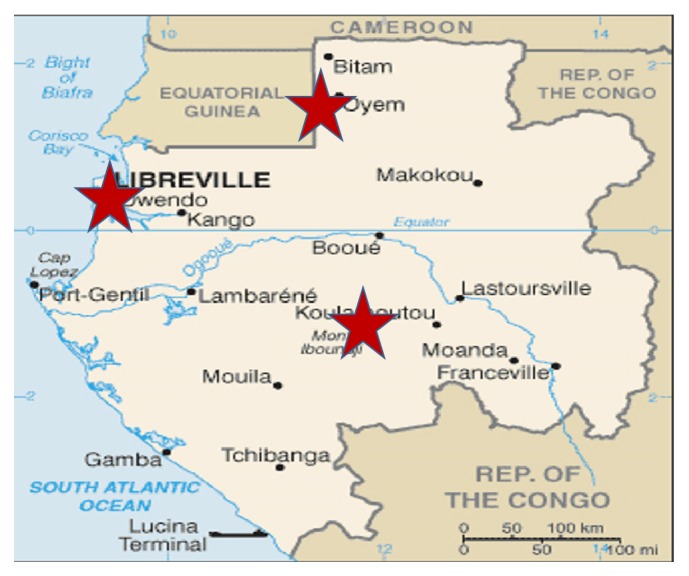
Study areas.

A cross-sectional study was carried out in three HIV care and treatment centers (CTC) located in Libreville, Oyem, and Koulamoutou between March 2015 and June 2016 (Figure 1).

Libreville, the capital city of Gabon brings together nearly a third of the population (579 577 inhabitants): Oyem and Koulamoutou, two rural cities located in Northern (60 000 inhabitants) and South Eastern (16 222 inhabitants) parts of the country, respectively.

With regard to the prevalence profile of HIV in the provinces hosting the study areas, Woleu-Ntem (Oyem) leads with 7.2%, Estuaire (Libreville) 3.7%, and Ogooué-Lolo (Koulamoutou) 3.0% in 2012. Malaria transmission is perennial in these areas and* P. falciparum* is the main species (96%). Malaria prevalence ranges from 24% in Libreville to 53% at Oyem (unpublished data). Patients living with HIV (PLHIV) attending their routine follow-up visits in HIV clinics were invited to participate in the study. Sociodemographic, clinical, and biological data were collected as described elsewhere [3].

### 2.2. Laboratory Procedures

#### 2.2.1. Malaria Diagnosis

Malaria diagnosis was performed by three different method tools.

First, LDH/HRP2, a rapid diagnostic test (SD BIOLINE, SD Standard Diagnostics Inc., South Korea), was performed according to the manufacturer's recommendations for the selection of positive samples. The results were communicated to the physicians for appropriate management. Afterwards, microscopy according to the Lambarene's method was performed, to confirm the presence of* P. falciparum* infection as previously described [26].

Otherwise, a nested polymerase chain reaction (PCR) assay targeting the* Plasmodium* small subunit ribosomal RNA (ssRNA) gene was assessed to detect* Plasmodium* in negative microscopic samples, as detailed elsewhere [22].

**Figure 2:**
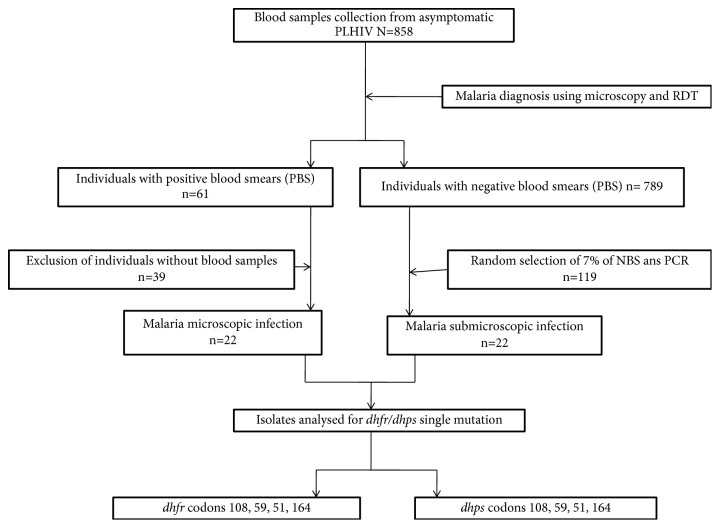
Study profile.

Malaria diagnosis was performed for all people living with HIV (n=858). Among them, 797 in the main study had a negative blood smear and 61 were infected with* P. falciparum*. According to the sample size calculation, as previously described [24], 7 percent of the 797 PLHIV with a negative blood smear was selected for the diagnosis of submicroscopic plasmodial infection. Submicroscopic infection was found in 10.1% of patients (12/119). Molecular genotyping was performed on* P. falciparum* isolates obtained by microscopy and PCR (Figure 2).

#### 2.2.2. Plasmodium falciparum Molecular Analysis

*P. falciparum *nucleic acid extraction was carried out with the QIAamp DNA Mini Kit (Qiagen, Germany), according to the manufacturer's instructions. Extracted DNA was stored at −20 °C until molecular analysis. SNPs at* Pfdhfr* 51, 59, 108, and 164 codons and* Pfdhps *437, 540, and 581 codons were detected. HRM method was performed as previously described [27, 28]. High resolution melting (HRM) analysis is an alternative method designed to investigate variance in nucleic acid sequences. HRM technique is the cheapest and sensitive compared to PCR-RFLP [27, 28].

HRM consists of an amplification of a target DNA and integration of a fluorochrome (sybre green) which emits fluorescence only when it is inserted between the two strands of the DNA. Through the process, DNA strands are separated during a progressive warming phase (from around 50°C up to around 95°C). Any given DNA sequence corresponds to a specific fluorescence evolution represented by a melting curve. Any change in the DNA sequence leads to a variation of the curve. Standard software is used to visualize melting peaks based on different melting temperatures, indicative of different base pairs, and for a comparison with controls.

### 2.3. Data Management and Statistical Analysis

Data were analyzed using STATVIEW 5.0 (SAS institute INC cary, USA). Patient characteristics were summarized using medians and interquartile ranges for continuous variables and proportions for categorical variables. Differences between groups were assessed using chi-squared or Fisher's exact tests if there were less than five expected values for proportions.* P *value of less than ≤0.05 was considered as significant. Prevalence of single or multiple allele was calculated in all isolates. Frequency of haplotype was calculated in the detected clones [8].

### 2.4. Ethical Approval

This study initiated by the Department of Parasitological Mycology was approved by the Ministry of Public Health and the Ethics Committee* (PROT No. 003/2016/SG/CNE)*. PLHIV participation was fully anonym and performed after informed consent was provided.

## 3. Results

### 3.1. PLHIV Characteristics

*Plasmodium falciparum* isolates from 34 participants were successfully genotyped. The median age of the patients was 43 (38-55) years. The median parasitemia was 518 (57-1562) p/*μ*l and 24.2% of patients took CTX. The median CD4 count was 313 [178-395]/mm^3^, higher in participants who took CTX compared to those who did not: 363 [283-591] / mm^3^ versus 257 [147-392] /mm^3^. More than half of the PLHIV used a bed net (12/22; 54.5%).

### 3.2. Prevalence of Mutations on* Pfdhfr* and* Pfdhps* Genes

**Figure 3:**
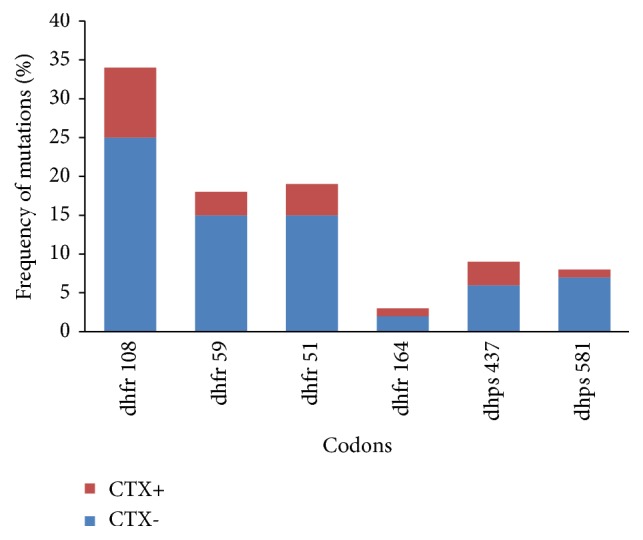
Frequency of single mutation detected on* Pfdhfr* and* Pfdhps* genes according to the use of cotrimoxazole.

*Pfdhfr *S108N mutation was detected in all isolates, while N51I and C59R mutations were found in 54.9% (19/34) and 52.9% (18/34) of the isolates, respectively. On* Pfdhps* gene, mutations were detected at codons A437G (26.5%; 9/34) and A581G (23.5%; 8/34)(*p*=0.2). No K540E mutation was detected. An unknown mutation was found at codon 164 (I164U) in three isolates, but not the I164L. According to CTX use, single mutations tended to be more frequent in isolates from patients not on CTX (Figure 3).

More than two-thirds of the isolates (70%; n= 24/34) harboured multiple mutations. Triple* Pfdhfr *mutations* (Pfdhfr *51I-59R-108N) were predominant (29.4%; n=10) while 17.6% (n=6) of the isolates carried a quadruple mutation (*p*=0.08). Quintuple mutation was detected in around 10% (n=3) of the samples. The* Pfdhfr *51I-59R-108N +* Pfdhps *437G + 581Gquintuple mutation was found in isolates from patients who did not use CTX (n=2) (Table 1).

### 3.3. Mixed Infections Frequency

The frequency of mixed infections ranged from 8.8% (n=3) to 23.5% (n=8) according to the codons on* dhfr* gene. It was the highest at codon 51 (23.5%; n=8) and codon 59 (20.5%; n=7). Mixed infections were found in three isolates at codon 108 and at codon 164, respectively. On* dhps* gene, they were found at codon 581 in 5.8% (n=2) of the isolates.

**Figure 4:**
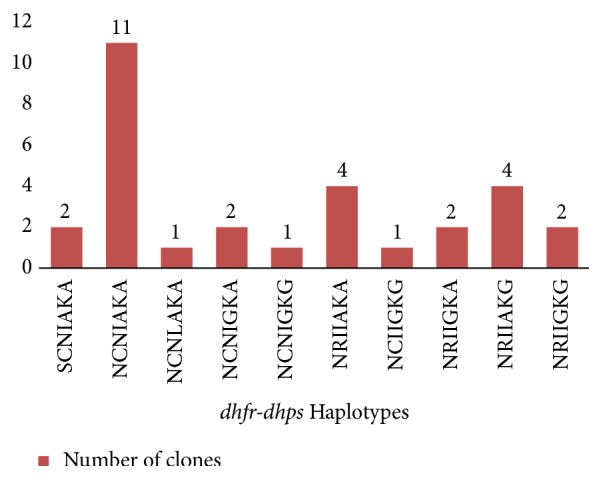
Distribution of the different haplotypes in the clones (n=30).

Among the isolates, 10 (10/34) carried mixed infections on at least 2 different codons. Haplotypes were analyzed in the remaining 24 isolates including those with multiple infections at one codon. Overall, 10 haplotypes were identified in the 30 clones detected (Figure 4). Two (n=2/30; 6.6%) clones had wild-type alleles at all codons. The most frequent haplotypes were those with a single mutation (**N**CNIAKA)(36%) and a quadruple mutation (23.3%) (**N**C**I**I**G**K**G**,** NRI**I**G**KA, and** NRI**IAK**G**).

## 4. Discussion

In Gabon, molecular markers of antifolates drug resistance have been investigated over the last 20 years in children and pregnant women before and after the introduction of IPTp-SP [9, 23, 24, 29]. This study is the first investigating the frequency of* Pfdhps *and* Pfdhfr *alleles in PLHIV adults who are or not on CTX prophylaxis in this country.

For the first time, polymorphism is found at* Pfdhps *codon 581 in almost a quarter (23.5%) of isolates from a subgroup of PLHIV in Gabon. A study reported few years ago A581G mutation in a single isolate collected from a patient living in Gabon although in the different studies performed in the country none has previously described this mutation in isolates from pregnant women and children [9, 24, 30, 31]. Recently, this mutation has been reported in low prevalence in the general population in Cameroon and Equatorial Guinea, two neighbouring countries of Gabon [15, 16, 32, 33]. Its detection presumably results from the technique used in all these studies, such as sequencing, multiplex PCR, or PCR-HRM, or may be due to the migratory population. Circulating parasites carrying this mutation may hamper preventive therapies with SP or CTX by an increase of the level of drug resistance of these parasites. Furthermore, the detection of this mutation could also be related to the area where the parasites populations have been collected for the first time (Koulamoutou) or after a long period (Oyem and Libreville) in regard to* P. falciparum* resistance investigations. Considering the area of origin of the isolates carrying the A581G mutation, 6 out of 8 of the isolates were from Libreville, a region where no A581G mutations were found in samples from children collected in 2014 and analyzed by sequencing (unpublished data). This mutation has also been found in isolates from PLHIV in Uganda after genes sequencing [34]. The commonly detected* Pfdhps *A437G has been found in 26.5% of the isolates. The frequency of this mutation is comparable to that obtained in isolates collected in Lambaréné in 1996 (28%). Nevertheless, it has over time gradually increased and may reach90% in some areas [25, 31, 35–38]. In contrast,* Pfdhps *K540E mutation which is not frequent in West and central Africa has not been detected in the isolates. At the time of IPTp-SP introduction in Gabon between 2005 and 2007the K540E mutation was absent or found in low prevalence [23, 29].* Pfdhfr* 108N mutation was detected in all isolates as previously reported in isolates (>96%) from children and pregnant women [24, 29]. Such high prevalence of* Pfdhfr* 108N was also found among isolates from PLHIV in Uganda [39, 40]. I164L mutation, which is rarely reported in central Africa, was also not detected in this study [15]. However, three isolates contained mixed infections at this codon with unknown mutant alleles. The identification of this mutation (I164U) is important and needs to be investigated in more samples and considering its association with other mutations. Indeed, such new mutation may contribute to increase in the level of pyrimethamine resistance, as observed with the known mutation (I164L) or inversely can have no impact on the development of drug resistance [10].

Multiple mutations, which are associated with* ex vivo* resistance to SP, were frequent (>70.0%) in the isolates. The frequency of isolates carrying a quintuple mutation combining the triple* Pfdhfr* and the double* Pfdhps* 437-581 mutation was comparable to the previously observed quintuple mutation including* Pfdhps* 437-540 mutation (6% versus 4%) [29]. This genotype is described in parasites considered as ‘fully resistant' and its presence also compromises the effectiveness of SP and CTX [13]. The SNP K540E which is usually included in the quintuple mutant tends to be replaced by A581G mutation. Its frequency is threefold higher than that obtained in Equatorial Guinea (2%) [15]. Among Kenyan and Ugandan PLHIV, a high frequency of the quintuple mutation (*Pfdhfr *51I-59R-108N +* Pfdhps *437G + 540E) was detected while the quintuple mutation (*Pfdhfr *51I-59R-108N +* Pfdhps *437G + 581G) was not [39, 40]. All quintuple mutants were found in individuals not exposed to CTX while double mutations were more frequent in isolates from participants exposed to CTX. Indeed, few PLHIV was under CTX at the time of the study; only PLHIV having a CD4 level <350 cell /*μ*L was under cotrimoxazole. In Ugandan PLHIV, no relation was found between the frequency of molecular markers of sulfamides and the intake CTX [39, 40]. Opportunistic infections are often the reason for discovering HIV infection, toxoplasmosis is the most common Ops, and the recommended treatment is CTX [41]. The use of SP as preventive therapy for pregnant women or children across Africa kept the pressure high after the withdrawal of SP as a curative measure. The spread of mutant* Pfdhfr *51I-59R-108N +* Pfdhps *437G + 581G genotypes could compromise the management of PLHIV as well as the efficacy of SP in IPTp or in combination with artemisinin derivatives used in Gabon. Despite the small sample size, these data highlight an important polymorphism in* Pfdhfr *and* Pfdhps* genes with the detection of new mutations or mutations that were not described previously in Gabon. The frequency of these genotypes in isolates from PLHIV, found here, suggests the need of more regular molecular monitoring of antimalarial drug resistance in these areas and more largely in uninvestigated areas of Gabon. The presence of the mutation* dhps581* and of a quintuple mutant should also be screened in the rest of the population as malaria parasites carrying mutations or not can circulate from individual to another.

## 5. Conclusion

In conclusion, this study highlights the presence of mutations related to high-level drug resistance, constituting a warning for the disease control purpose.* Pfdhps *A581G mutation, rarely described in central Africa as well as new quintuple mutation was found for the first time in PLHIV in Gabon. The nonnegligible frequency of these genotypes in the parasite population requires a regular surveillance of antifolates drug resistance in populations exposed to this drug. Taking CTX for the treatment of these infections was not associated with the occurrence of super-resistant genotypes. These genotypes may have an impact on malaria control strategies and prevention.

## Figures and Tables

**Table 1:** Distribution of multiple *Pfdhfr/Pfdhps* mutation according to cotrimoxazole prophylaxis.

*Multiple mutation* ^*§*^	All	On CTX∗	Not on CTX
	(N=34)	(N=8)	(N=26)

*Double mutation, n(%)*	*3(8.8)*	*2(25.0)*	*1(3.8)*
S108N/I164U^+^	1(2.9)	1(12.5)	0 (0.0)
S108/A437G	2(5.8)	1(12.5)	1(3.8)
*Triple mutation,n(*%)	*12(35.3)*	*2(25.0)*	*10(38.5)*
S108N/N51I/C59R	10 (29.4)	2(25.0)	8(30.8)
*Triple pfdhfr-pfdhps*			
S108N/I164L/A581G	1(2.9)	0(0.0)	1(3.8)
S108N/A437G/A581G	1(2.9)	0(0.0)	1(3.8)
*Quadruple mutation,n(*%)	*6(17.6)*	*2(25.0)*	*4(15.4)*
*Quadruple pfdhfr/pfdhps*			
S108N/N51I/C59R/A437G	2 (5.8)	1(1.25)	1(3.8)
S108N/N51I/C59R/A581G	3 (8.8)	0(0.0)	3(11.5)
S108N/N51I/A437G/A581G	1(2.9)	1(12.5)	0(0.0)
S108N/N51I/C59R/A437G			
*Quintuple mutation *	*3(8.8)*	*0*(0.0)	*3(11.5)*
S108N/N51I/C59R/A437G/A581G	2 (5.9)	0	2(7.7)
S108N/N51I/C59R/I164U^+^/A437G	1(2.9)	0	1(3.8)

^§^Isolates carrying mutations at two or more codons. ^**+**^Unknown mutant genotype. ∗Two isolates carried SNP at codon S108N.

## Data Availability

The data used to support the findings of this study are available from the corresponding author upon request.
